# Evaluation of the validity and reliability of the 10-meter walk test using a smartphone application among Japanese older adults

**DOI:** 10.3389/fspor.2022.904924

**Published:** 2022-10-04

**Authors:** Yoshinobu Saito, Sho Nakamura, Ayumi Tanaka, Ryo Watanabe, Hiroto Narimatsu, Ung-il Chung

**Affiliations:** ^1^Faculty of Sport Management, Nippon Sport Science University, Yokohama, Japan; ^2^Center for Innovation Policy, Kanagawa University of Human Services, Kawasaki, Japan; ^3^Cancer Prevention and Control Division, Kanagawa Cancer Center Research Institute, Yokohama, Japan; ^4^Graduate School of Health Innovation, Kanagawa University of Human Services, Kawasaki, Japan; ^5^Division of Health Promotion, Fujisawa City Health and Medical Foundation, Fujisawa, Japan; ^6^Department of Bioengineering, Graduate School of Engineering, The University of Tokyo, Tokyo, Japan

**Keywords:** walking speed, smartphone application, mobile health (M-Health), public health, health promotion

## Abstract

**Objective:**

Maintaining or improving regular walking speed can help extend healthy life expectancy and prevent frailty. The evaluation of walking speed can help individuals meet their daily exercise goals; therefore, it may be beneficial as a public health policy for residents to measure and evaluate their walking speed easily. This study aimed to verify the validity and reliability of a smartphone application for the 10-m walk test, measured in the general population.

**Methods:**

The study participants were men (*n* = 20) and women (*n* = 20) aged 65–85 years. The 10-m walk tests were performed at the usual walking speed, using the stopwatch function of a newly developed smartphone application. A total of three 10-m walk tests were performed simultaneously with the study participants and professional fitness instructors to evaluate the criterion-related validity and the test-retest reliability.

**Results:**

A strong positive correlation was found in the criterion-related validity by the study participants and professional staff for the average of the three trials {*r* = 0.961 [95% confidence interval (CI) = 0.927, 0.979]}. The results revealed good reliability, with an intraclass correlation coefficient of 0.712 (95% CI = 0.571, 0.823).

**Conclusion:**

The smartphone application walking speed measurement method can be widely used by the general public and is useful for health promotion.

## Introduction

The 10-m walk test is widely used in preventive care and rehabilitation; it is evaluated by measuring speed and counting the number of steps taken within a given time. The measurement method includes walking a certain section set on a flat road at the usual or maximum speed, after which the walking time and number of steps taken are measured. Criterion-related validity has shown significant correlations in measurement methods (10 m and 4 m, stopwatch, and automatic timer) (1) and lower extremity muscle strength (sit-to-stand test) (2). Test-retest reliability by trained staff has also been high (1, 3).

Maintaining or improving usual walking speed is associated with reduction or prevention of mortality, hospitalization, activities of daily living ability, and cognitive decline (4, 5). Usual walking speed is used as a criterion for assessing frailty and sarcopenia, which are associated with a high risk of care dependency. In Asian countries, sarcopenia is defined as a low walking speed of < 1.0 m/s (6). The minimum clinically meaningful change in normal walking speed is 0.05 m/s, which is an indicator of the effect of treatment on an individual, while a substantial change is reported at 0.10 m/s (7).

The evaluation of walking speed is beneficial for extending healthy life expectancy; therefore, it may be a helpful public health policy for residents to easily measure and evaluate it. Furthermore, implementing a method that can be easily performed using a smartphone application is expected to be quickly adopted in a healthcare set-up.

In response to this situation, the ME-BYO index was implemented in Kanagawa Prefecture, one of the biggest local governments in Japan, that measured the ME-BYO in the health management application, My ME-BYO Record, in March 2020 (8). ME-BYO is a concept that does not consider health and sickness as two separable conditions; it is a concept that covers the entire process of continuous change in the physical and mental condition between health and sickness (8).

The ME-BYO index visualizes an individual's current state of the ME-BYO and future disease risk in numerical form. It comprehensively quantifies the domains of lifestyle, physical function, cognitive function, mental health, and stress. The ME-BYO index was developed based on evidence and discussion among experts. Specific evaluation items included gender, age, body mass index, systolic blood pressure (lifestyle), Mini-Cog (cognitive function) (9), locomotive function (10), walking speed (physical function), and mind-monitoring system (mental health and stress) (11).

To assess walking speed, a 10-m walk test (12) with a stopwatch function was adopted. This study aimed to verify the validity and reliability of the 10-m walk test measured by the general population.

## Methods

### Study participants

Forty target participants (20 men and 20 women) aged 65–85 years were included in the study. They attended health promotion facilities on their own and performed exercises. In the sample size design, assuming a significance level of 5%, power of 80%, and effect size of 0.5, the required number of participants was 29. The final number of study participants was set at 40, assuming dropouts and multiple applicants signed up at the same time.

This study was approved by the Research Ethics Committee of the Graduate School of Health Innovation, Kanagawa University of Human Services (approval no. Hodai 30–011). The purpose and content of the study were explained to the study participants, and written informed consent was obtained from them before the study was conducted.

### Measurement items

#### The 10-M walk test using the application by the study participants

The study participants measured their walking speed using a smartphone equipped with the ME-BYO index application for research (Supplementary Figure 1). The research application was designed to store data locally on the device. The ME-BYO index for general use is implemented in the My ME-BYO Record application, which can be downloaded from the App Store.

For the 10-m walk test, the participants followed the on-screen instructions to walk 10 m at their usual speed, and the walking time was measured. The site was a flat indoor area with 4-m preliminary and deceleration zones. The time taken for the 10-m walk was measured using the stopwatch function in the application. Before starting the measurement, the participants waited in a stationary standing position at the starting point of the preliminary zone. The participants walked to the goal point of the deceleration path (18 m) without stopping at the end of 10 m.

Measurements were taken by tapping the screen as soon as either toe crossed the line between the start and end lines. If the speed was faster or slower than the usual walking speed, the participant was asked to confirm and re-measure. Walking time was measured to the nearest hundredth of a second. The walking speed was calculated by dividing the distance (10 m) by the walking time (m/s).

#### The 10-M walk test by professional staff

To verify the criterion-related validity, the measurements by the professional staff and the study participants were simultaneously conducted. The measurement was conducted by a health fitness instructor with sufficient knowledge and experience in using the stopwatch method, which is widely used in clinical practice (ALBA PICCO Standard, manufactured by SEIKO, Tokyo, Japan). The timing of starting and ending the stopwatch was the same as that of the study participants.

The staff walked diagonally behind the study participants to avoid guiding the study participants. The distance between the study participant and the instructor was not too large so that the instructor could immediately support the participant in case of a fall and did not interfere with the study participant's walking.

#### Five-time sit-to-stand test

The five-time sit-to-stand test (13) required participants to stand up from and sit down on a slightly padded 42-cm high armless chair as quickly as possible five times. Participants folded their arms across their chests and were instructed to stand up completely and make firm contact with the chair when sitting. The commencement time began on the command “go” and ceased when the participants sat after the fifth stand-up. Participants were allowed a practice trial of one to two repetitions before the test trial. The measurements were taken by a health fitness instructor who used a stopwatch to measure the time to 1/100th of a second, rounded off to the nearest tenth of a second.

#### Basic attributes

The following information was obtained for the measurement items of the ME-BYO index application: sex, date of birth (age), height, weight, and blood pressure. These items were measured before the 10-m walk test. Data on educational attainment, working status, and living arrangement were obtained using a questionnaire.

### Statistical analysis

The normality of the walk test was analyzed using the Shapiro–Wilk test. The criterion-related validity of the walk test was assessed by Pearson's correlation coefficient for the speed (m/s) of the 10-m walk in the ME-BYO index application performed by the study participants and health fitness instructors. The second value and average of the three values were employed to validate the walk test, as in previous studies (1). Test-retest reliability was assessed using intraclass correlation coefficient (ICC) and 95% confidence intervals (CIs). For test-retest reliability, the values of all three tests were used. For the statistical analysis, SPSS version 27 (IBM, Tokyo, Japan) software was used. The significance level was set at 5%.

## Results

### Basic attributes

This study included 40 participants (20 men and 20 women) with a mean age (standard deviation) of 74.9 (5.2) years. Other participant characteristics are shown in Table 1.

**Table 1:** Characteristics of the study participants.

	**Men (*****n*** = **20)**		**Women (*****n*** = **20)**		**Total (*****n*** = **40)**	
**Age, years**	75.3	(5.3)	74.6	(5.2)	74.9	(5.2)
**Educational attainment, n (%)**						
< 13 years	8	(40.0)	10	(50.0)	18	(45.0)
≥13 years	12	(60.0)	9	(45.0)	21	(52.5)
Missing	0	(0)	1	(5.0)	1	(2.5)
**Working status, n (%)**						
Working with income	6	(30.0)	5	(25.0)	11	(27.5)
Not working	14	(70.0)	14	(70.0)	28	(70.0)
Missing	0	(0)	1	(5.0)	1	(2.5)
**Living arrangement, n (%)**						
With others	19	(95.0)	16	(80.0)	35	(87.5)
Alone	1	(5.0)	3	(15.0)	4	(10.0)
Missing	0	(0)	1	(5.0)	1	(2.5)
**Height, cm**	166.2	(7.7)	153.2	(6.4)	159.7	9.6
**Body weight, kg**	63.9	7.7	49.8	4.8	56.8	9.6
**Body mass index, kg/m** ^**2**^	23.1	(1.7)	21.2	(2.1)	22.2	(2.1)
**Systolic blood pressure, mmHg**	138.4	(13.3)	131.8	(18.7)	135.1	(16.3)
**Diastolic blood pressure, mmHg**	75.7	(10.5)	71.4	(9.5)	73.5	(10.1)
**Average walking speed (Participants), m/sec**	1.40	(0.1)	1.46	(0.1)	1.43	(0.1)
**Average walking speed (Health fitness instructor), m/sec**	1.40	(0.1)	1.46	(0.1)	1.43	(0.1)
**5-times sit-to-stand test, sec**	6.9	(2.2)	5.1	(0.9)	6.0	(1.9)

Mean (standard deviations).

The mean values (standard deviations) of the three trials of the participants and the health fitness instructor in the 10-m walking speed were 1.43 (0.1) m/s and 1.43 (0.1) m/s for men and women, respectively. All walk tests were normal distribution.

### Validity and reliability of the 10-M walk test by study participants and professional staff

The results of the validity and reliability of the 10-m walk test are shown in Table 2. As a result of the criterion-related validity of the walking speed of the ME-BYO index application measured by the study participants and health fitness instructors, Pearson's correlation coefficient was *r* = 0.862 (95% CI = 0.753, 0.925), *P* < 0.001 for the second trial, and *r* = 0.961 (95% CI = 0.927, 0.979), *P* < 0.001 for the average of the three trials, indicating a significantly strong positive correlation.

**Table 2:** Validity and reliability of the 10-meter walk test by study participants and professional staff.

	**Criterion-related validity**			**Test-retest reliability**		
	***r***	**95% CI**	***P***	**ICC**	**95% CI**	***P***
Walking speed: second trial	0.862	0.753, 0.925	< 0.001	0.712	0.571, 0.823	< 0.001
Walking speed: average of three trials	0.961	0.927, 0.979	< 0.001			

Criterion-related validity: Pearson's correlation coefficient. Test-retest reliability: ICC (1, 1). ICC, Intraclass correlation coefficient; CI, confidence interval.

The ICC (95% CI) for test-retest reliability was 0.712 (0.571, 0.823), *P* < 0.001, indicating moderate reliability.

### Validity of the 10-M walk test measured by study participants and five-time sit-to-stand test measured by professional staff

The criterion-related validity of the walking speed of the ME-BYO index application measured by the research participants and the five-time sit-to-stand test measured by health fitness instructors was examined. Pearson's correlation coefficient was *r* = −0.572 (95% CI = −0.750, −0.317), *P* < 0.001 for the second trial, and *r* = −0.579 (95% CI = −0.754, −0.326), *P* < 0.001 for the average of the three trials, indicating a significant positive correlation.

## Discussion

This study examined the validity and reliability of a method in which the general population underwent the 10-m walk test, which is generally conducted at the usual walking speed, using the stopwatch function of a smartphone application. A strong positive correlation was found in criterion-related validity with the method measured by professional staff (health fitness instructors). In previous studies, a moderate correlation was found in the validity of the five-times sit-to-stand test (2), which has been adopted as an evaluation index for sarcopenia (6) and the World Health Organization Integrated care for older people (WHO ICOPE) guidelines (14); this method also has good reliability. In locations with no space to measure the walk test, the five-time sit-to-stand test was an option. Furthermore, in the Bland–Altman analysis, the agreement between professional staff and participants was high, and the systematic error was within range (Supplementary Figure 2). Subgroup analysis based on sex and blood pressure also showed similar results (Supplementary Tables 1, 2). These results suggest that the smartphone application measurement method can be widely used by the general public and is useful for health promotion.

The validity and reliability of the 10-m walk test and the 4-m walk test, measured by professional staff, have been studied. The validity and reliability of these studies were comparable to that of the present study (1). The reliability of the 4-, 6-, and 10-m walk tests with the usual pace measured by trained staff in older adults (3) was also similar to that of this study (ICC 0.72–0.90). Considering that our results were comparable to previous studies and that this study was conducted among the general population, it suggests that health promotion activities for residents can be expanded using the measurement methods used in this study. Moreover, high validity and reliability were confirmed among the older adults, and the results can be generalized to the younger generation, most of whom use smartphones.

Conversely, the 10-m walk test requires a place for measurement. When this application is disseminated, it is necessary to improve the environment where the measurements are obtained so that it can be conducted in community parks and exercise facilities. Furthermore, an application for the automatic measurement of walking speed with a patented technology using the global positioning system function has been implemented (15), and it is necessary to consider using it in conjunction with such technology.

One of the limitations of this study is that it did not involve random sampling and only included healthy, older adults with relatively high walking speeds. Therefore, caution should be exercised when implementing the insights generated in this study on residents with low cognitive function and locomotive impairments.

In conclusion, we confirmed the validity and reliability of a 10-m walk test application measured by the residents using the stopwatch function of a smartphone application. Moreover, the results were equally favorable for sex and the population with hypertension. This application has sufficient scientific validity to be measured by residents and can be used as motivation to maintain and improve health behavior.

## Data availability statement

The raw data supporting the conclusions of this article will be made available by the authors, without undue reservation.

## Ethics statement

The studies involving human participants were reviewed and approved by Research Ethics Committee of the Graduate School of Health Innovation, Kanagawa University of Human Services (approval no. Hodai 30-011). The patients/participants provided their written informed consent to participate in this study.

## Author contributions

YS: conceptualization, methodology, formal analysis, investigation, data curation, writing–original draft, writing–review and editing, and project administration. SN: conceptualization, methodology, and writing–review and editing. AT: investigation and writing–review and editing. RW: conceptualization and writing–review and editing. HN: conceptualization, methodology, writing–review and editing, supervision, project administration, and funding acquisition. UC: conceptualization, writing–review and editing, supervision, and funding acquisition. All authors have read and approved the final manuscript.

## Funding

This study was supported by the ME-BYO index project of the Kanagawa Prefecture and Grants-in-Aid for the Japan Society for the Promotion of Science (JSPS) KAKENHI Grant [No. 16H06277 (CoBiA)] from the Japanese Ministry of Education, Culture, Sports, Science, and Technology.

## Conflict of interest

The authors declare that the research was conducted in the absence of any commercial or financial relationships that could be construed as a potential conflict of interest.

## Publisher's note

All claims expressed in this article are solely those of the authors and do not necessarily represent those of their affiliated organizations, or those of the publisher, the editors and the reviewers. Any product that may be evaluated in this article, or claim that may be made by its manufacturer, is not guaranteed or endorsed by the publisher.

## Abbreviations

ICC: intraclass correlation coefficient
CIs: confidence intervals
WHO ICOPE: World Health Organization Integrated care for older people.
