# Supplementary material for: Evaluation of the validity and reliability of the 10-meter walk test using a smartphone application among Japanese older adults
Source: Front Sports Act Living. 2022 Oct 4;4:904924. doi: 10.3389/fspor.2022.904924 (PMC9576938; doi:10.3389/fspor.2022.904924)
Supplement: Supplementary file 1 [file Data_Sheet_1.docx]

Supplementary Material


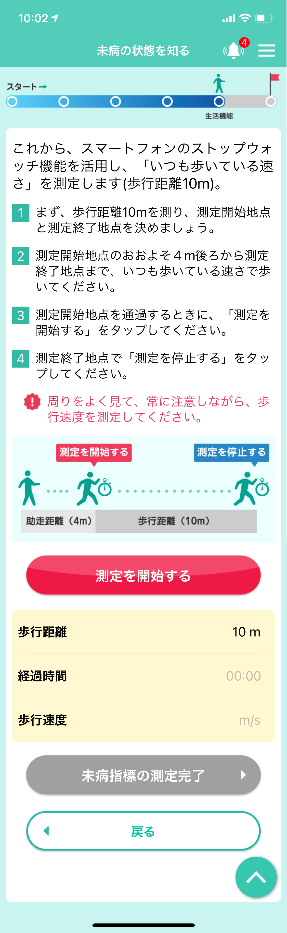


**Figure S1. Screenshot of the 10-meter walk test application.**


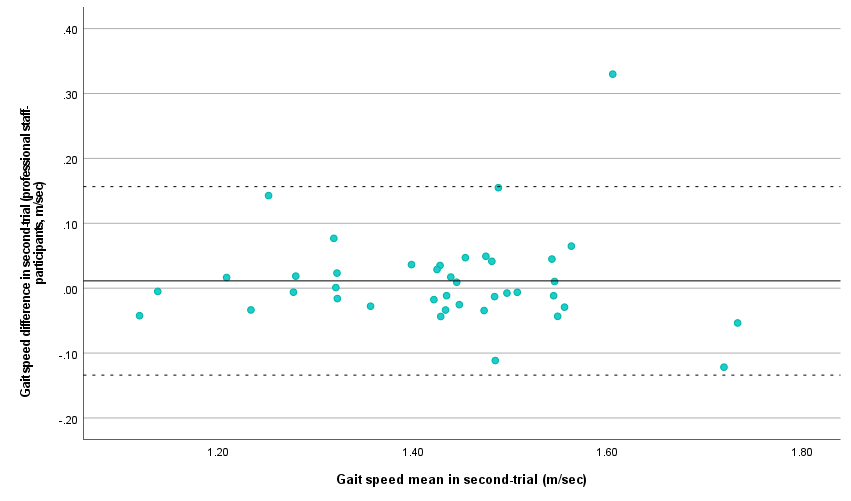


**Figure S2. Bland-Altman plots representing comparisons between professional staff-participants gait speed assessments obtained using a stopwatch for second-trial gait speed.**

The solid line represents the mean difference in gait speed between the 2 walking tests, with the dashed lines representing the upper and lower 95% limits of agreement, mean ± 1.96 SD.

SD; Standard Deviation

**Table S1. Subgroup analysis based on sex in validity and reliability of the 10-meter walk test by study participants and professional staff**

|  | | Criterion-related validity | | | Test-retest reliability | | |
| --- | --- | --- | --- | --- | --- | --- | --- |
|  |  | *r* | 95% CI | *P* | ICC | 95% CI | *P* |
| Men  （n= 20） | Gait speed: second trial | 0.902 | 0.765, 0.961 | <0.001 | 0.680 | 0.458, 0.844 | <0.001 |
|  | Gait speed: average of three trials | 0.967 | 0.916, 0.987 | <0.001 |  |  |  |
| Women  （n= 20） | Gait speed: second trial | 0.823 | 0.598, 0.928 | <0.001 | 0.734 | 0.535, 0.873 | <0.001 |
|  | Gait speed: average of three trials | 0.953 | 0.884, 0.982 | <0.001 |  |  |  |
| Criterion-related validity: Pearson's correlation coefficient  Test-retest reliability: ICC (1,1)  ICC: Intraclass correlation coefficient  CI: confidence interval | | | | | | | |

**Table S2. Subgroup analysis based on blood pressure in validity and reliability of the 10-meter walk test by study participants and professional staff**

|  | | Criterion-related validity | | | Test-retest reliability | | |
| --- | --- | --- | --- | --- | --- | --- | --- |
|  |  | *r* | 95% CI | *P* | ICC | 95% CI | *P* |
| Persons not applicable to high blood pressure （n= 20） | Gait speed: second trial | 0.803 | 0.591, 0.911 | <0.001 | 0.684 | 0.485, 0.834 | <0.001 |
|  | Gait speed: average of three trials | 0.958 | 0.905, 0.982 | <0.001 |  |  |  |
| Persons with high blood pressure （n= 20） | Gait speed: second trial | 0.961 | 0.889, 0.987 | <0.001 | 0.772 | 0.565, 0.904 | <0.001 |
|  | Gait speed: average of three trials | 0.965 | 0.900, 0.988 | <0.001 |  |  |  |
| Criterion-related validity: Pearson's correlation coefficient  Test-retest reliability: ICC (1,1)  ICC: Intraclass correlation coefficient  CI: confidence interval  High blood pressure criteria (World Health Organization): Systolic blood pressure ≥140 mmHg and/or Diastolic blood pressure ≥90 mmHg | | | | | | | |
